# Activity of Pt(II) and Ru(III) Triazolopyrimidine Complexes Against Parasites of the Genus *Leishmania*, *Trypanosomas* and *Phytomonas*

**DOI:** 10.1155/MBD.2001.119

**Published:** 2001

**Authors:** Juan M. Salas, Miguel Quirós, Mohammad Abul Haj, Rosa Magán, Clotilde Marín, Manuel Sáchez-Moreno, René Faure

**Affiliations:** 1 Departamento de Química Inorgánica Facultad de Ciencias Universidad de Granada Granada 18071 Spain; 2 Depatamento de Parasitología Facultad de Ciencias Universidad de Granada Granada 18071 Spain; 3 LICAS Bâtiment 305 Université Claude Bernard Lyon 1 Villeurbanne 69622 France

## Abstract

The synthesis and characterization of two Pt(II) Complexes with the isomeric ligands 4,5-dihydro-5-oxo- [1,2,4]triazolo-[ 1,5-a]pyrimidine (5HtpO) and 4,7-dihydro-7-oxo-[ 1,2,4]-triazolo-[ 1,5-a]pyrimidine (7HtpO) are described, as well as a Ru(III) complex with 7HtpO. The crystal structure of *cis*-[PtCl_2_(7HtpO)_2_].2H_2_O has been solved by X-ray diffraction analysis. *In vitro* activity of the new  isolated complexes against the epimastigote form of *T. cruzi*, procyclic form of *T. b. brucei* and promastigote form of *L. donnovani* and *P. characias* has also been studied. The three complexes markedly affect the growth of the parasites and none of them shows cytotoxicity against macrophage of the J774.2 line at the heaviest dosages used.


### ACTIVITY OF Pt(II) AND Ru(III) TRIAZOLOPYRIMIDINE COMPLEXES AGAINST PARASITES OF THE GENUS *LEISHMANIA*, *TRYPANOSOMAS* AND *PHYTOMONAS*

Juan M. Salas,[1] * Miguel Quirós,[1] Mohammad Abul Haj,[1] Rosa Magán,[2] Clotilde Marín,[2] Manuel Sánchez-Moreno[2] and René Faure[3]

[1] Departamento de Química Inorgánica, Facultad de Ciencias. Universidad de Granada. 18071 Granada, Spain
[2] Departamento de Parasitología, Facultad de Ciencias. Universidad de Granada. 18071 Granada, Spain
[3] LICAS, Bâtiment 305, Université Claude Bernard Lyon 1, 69622 Villeurbanne, France

**Abstract**

The synthesis and characterization of two Pt(II) complexes with the isomeric ligands 4,5-dihydro-5-oxo-[1,2,4]triazolo-[1,5-a]pyrimidine (5HtpO) and 4,7-dihydro-7-oxo-[1,2,4]-triazolo-[1,5-a]pyrimidine (7HtpO) are described, as well as a Ru(III) complex with 7HtpO. The crystal structure of *cis*-$[PtCl_2(7HtpO)_2].2H_2O$ has been solved by X-ray diffraction analysis. *In vitro* activity of the new isolated complexes against the epimastigote form of *T. cruzi*, procyclic form of *T. b. brucei* and promastigote form of *L. donnovani* and *P. characias* has also been studied. The three complexes markedly affect the growth of the parasites and none of them shows cytotoxicity against macrophage of the J774.2 line at the heaviest dosages used.

### INTRODUCTION

The members of the family Trypanosomatidae are the etiological agents of numerous diseases which afflict not only humans but also animals and plants. Examples include American trypanosomiasis, or Chagas' disease, which is caused by *Trypanosoma cruzi*,[1] African trypanosomiasis, caused by two strains of *Trypanosoma brucei*,[2] *Leishmania donovani*, inflicting visceral leishmaniasis or Kala-azar and *Phytomonas characias,* which attacks the lactiferous plant *Euphorbia characias,* resulting in defoliation, deformation, chlorosis and atrophy.[3] Even in the 21st century, despite the development of efficient drugs against these protozoan parasites, the eradication of such diseases remains seriously deficient. The administration of these drugs entails a number of drawbacks, such as variable efficacy, normally long treatment often with toxic side-effects, parenteral administration for some diseases and the appearance of resistant strains.[4-6] Another problem is that the development of new drugs requires heavy financial investment from the industry and the areas most in need of these drugs are economically depressed, and therefore the industry takes little interest in developing these drugs. The search for new compounds is, therefore, urgent.

Choosing [1,2,4]triazolo[1,5-a]pyrimidine derivatives for our studies is based on the fact that these ligands could be considered as mimetic of purines. Different researchers have studied them as chemotherapeutic agents, finding different interesting properties, such as the growth inhibition of some microorganisms.[7] With regard to their antitumoural activity, studies have been made for various cell lines such as the breast cancer MDA/MB-231.[8] Their potential in the treatment of neurodegenerative disorders such as Parkinson's disease has also been tested.[9]

The coordination chemistry of these ligands has been extensively studied in recent years.[10,11] They have, as potential donors, three endocyclic nitrogen atoms at positions 1, 3 and 4, N(3) being the preferred metal binding site in the monodentate mode (see Figure 1 for the numbering scheme used for these ligands). Another possibility found in several cases is the bridging N(3)-N(4) mode giving dinuclear metal complexes.[12,13]

We have shown, in previous works, the biological activity of some metal complexes of [1,2,4]triazolo[1,5-a]pyrimidine derivatives. In this way, we have recently tested the effect of several metal complexes of 4,7-dihydro-5-methyl-7-oxo [1,2,4]-triazolo-[1,5-a]pyrimidine (HmtpO) against *Phytomonas Staheli* (promastigote form), *Trypanosoma cruzi* and *Leishmania donovani*.[14-15] Some of them proved capable of inhibiting the growth of these flagellates at dosages of 50 μM after 24 h of activity, inhibiting the synthesis of macromolecules (DNA, RNA and proteins) by the parasites and inducing severe damage in their ultrastructure.[14]

With this new work, we seek to advance one step further in the development of effective chemotherapy against members of the family Trypanosomatidae by testing three newly synthesized metal complexes (two of Pt(II) and one of Ru(III)) of triazolopyrimidine derivatives on the epimastigote forms of *T. cruzi*, procyclic forms of *T. b. brucei* and promastigote forms of *L. donovani* and *P. characias*, determining the effect of these complexes on the *in vitro* growth of the parasites as well as the possible toxicity of these treatments at macrophage level.

### MATERIALS AND METHODS

*Chemicals*

Hydrated ruthenium(III)chloride was supplied by Johnson Matthey and potassium tetrachloroplatinate(II) by Sigma-Aldrich Chem. and were used without further purification. The isomeric ligands 4,5-dihydro-5-oxo[1,2,4]triazolo[1,5-a]pyrimidine (5HtpO) and 4,7-dihydro-7-oxo [1,2,4]triazolo[1,5-a]pyrimidine (7HtpO) were synthesized using previously described methods.[16]

*Synthesis of the metal complexes*

The platinum(II) complexes *cis*-$[PtCl_2(7HtpO)_2].2H_2O$ (**1**) and $[PtCl_2(5HtpO)_2].2H_2O$ (**2**) were prepared by mixing two solutions in HCl 1N, 10 mL each, the first one containing 2 mmol of $K_2[PtCl_4]$ and the second one containing 4 mmol of 7HtpO or 5HtpO. The resulting solutions were left to evaporate at room temperature and yellow crystals, suitable for X-ray analysis in the case of **1**, were obtained after a few days, whereupon they were collected by filtration and air dried. Calculated elemental analysis for $C_{10}H_{12}O_4N_8Cl_2Pt$: C, 20.92; H, 2.11; N, 19.51%. Found: C. 20.75; H, 2.09; N. 19.24% (**1**) and C, 20.91; H, 2.11; N. 19.35% (**2**).

The ruthenium(III) complex $RuCl_3(7HtpO)_2.2H_2O$ (**3**) was obtained by mixing two solutions, 25 mL each, one containing 6 mmol of ruthenium(III) chloride in water an the other containing 6 mmol of 7HtpO in 1N HCl. The resulting solution was heated and stirred for 1h. After cooling a brown dark precipitate was isolated by filtration, washed with ethanol and air dried. Calculated elemental analysis for $C_{10}H_{12}O_4N_8Cl_3Ru$: C, 23.29; H, 2.35; N, 21.75%. Found for **3**: C, 23.01; H, 2.68; N, 21.65%.

Table 1. Crystal data for **1**

| | |
|---|---|
| Molecular formula | $C_{10}H_{12}Cl_2N_8O_4Pt$ |
| Formula weight | 574.27 |
| Crystal system | Monoclinic |
| Space group | P $2_1$/n |
| a, Å | 10.046(2) |
| b, Å | 13.354(3) |
| c, Å | 13.128(3) |
| b, ° | 112.08(3) |
| V, $Å^3$ | 1631.9(6) |
| Z | 4 |
| $D_{calc}$, g $cm^{-3}$ | 2.337 |
| m(MoKα), $mm^{-1}$ | 8.962 |
| Unique reflections | 3734 |
| wR2 | 0.1316 |
| R (F>4σ(F)) | 0.0475 |
| Final ΔF, $eÅ^{-3}$ | -3.663 - 6.850 |
| Deposition number | CCDC 158297 |

*Instrumentation*

Elemental analysis were performed in a Fisons Instruments EA-1008 analyzer and $^1$H-NMR spectra were recorded for DMSO-$d_6$ solutions in a Bruker AM300 equipment, both at the Centre of Scientific Instrumentation of the University of Granada.

*X-ray determination*

A colourless prismatic single crystal of 0.3 x 0.05 x 0.02 mm was mounted in a Nonius Kappa CCD diffractometer with MoKα radiation (λ = 0.71073 Å). The crystal data and the most relevant experimental parameters are reported in Table 1. Structure solution and refinement was performed by standard crystallographic procedure. The program SHELX-97[17] was used for the refinement.

*Biological Assays*

The strain of *T. cruzi* was isolated in 1972 from a human clinical case in Venezuela (Malariology Institute, Maracay, Venezuela). Epimastigote forms were obtained in biphasic medium (NNN supplemented with MEM and 20% IFBS) and afterwards reseeded in a monophasic culture (LTM), following the method of Ruiz-Perez et al.[18] The procyclic forms of *T. b. brucei* used in the experiment came from a stock 120.499, kindly given by Fred R. Opperdoes, from the Search Unit for Tropical Diseases at the International Institute of Cell and Molecular Pathology in Brussels (Belgium). These forms were cultured *in vitro* at 28°C in plastic Roux flasks, seeding an inoculate of 5 x $10^4$ cells per mL into 3 mL of culture medium (SDM-79 with 10% inactivated SBF added).

The *L. donovani* strain L133 (Leishmania Reference Centre, Jerusalem) was maintained by culture in NNN medium (GIBCO) and 20% IFBS (LTM). Afterwards, promastigote forms were obtained by reseeding in monophasic culture, TC-199 (GIBCO) supplemented with 30% IFBS. *P. characias*, originally isolated from *Euphorbia characias*, was generously provided by Dr. M.Dollet. The flagellates were cultured in Grace's medium at 28°C, following Fernández-Becerra et al.[19]

All the forms were taken at the exponential growth phase, centrifuged at 1500 g for 10 min and 4°C and resuspended in medium. The number of parasites was quantified using an haemocytometer chamber and adjusted to $10^6$/ml.

Mouse macrophages J-774A.1 (ECACC85011428) were cultured in RPMI 1640 medium (GIBCO) supplemented with 2mM L-glutamine and 10% IFBS at pH 7.2.

The three metal complexes assayed were dissolved in DMSO, and tested at 1, 20, 50 and 100 μM in culture medium with 0.01% DMSO final concentration. Controls and assays were prepared including 0.01% DMSO. Assays were performed in Roux flasks with a final volume of 10 ml and an original density of $1x\ 10^6$ cells/ml. The cultures were maintained for 24, 48 and 72 h at 28°C. For each time and dosage tested, five replicates and corresponding controls were quantified using a Neubauer chamber.

### RESULTS AND DISCUSSION

*NMR spectra*

The $^1$H-NMR spectra of DMSO-$d_6$ solutions of both platinum complexes, if recorded immediately after dissolving the compounds, display a set of signals of a majoritary species, presumiblely the same present at the solid state. The positions (in ppm.) and assignments of the signals are as follows:

Compound **1**: 6.10 (d, H(6)), 8.08 (d, H(5)), 8.56 (s, H(2)), J(H(5)-H(6)) = 7.6 Hz. H(4) not observed.

Compound **2**: 6.52 (d, H(6)), 8.64 (s, H(2)), 8.81 (d, H(7)), J(H(6)-H(7)) = 7.4 Hz. H(4) not observed.

All these signals are downfield shifted with respect to their positions in the free ligands,[16] the highest shift being that of H(2), in agreement with N(3) coordination to the platinum atom.

If the DMSO-$d_6$ solutions of the complexes are left for several hours and spectra are taken again, we can see that the intensity of the signals assigned to the Pt complexes decrease at the same time that new signals appear and grow, at positions identical to those of the free ligands. This indicate us that the complexes are unstable in DMSO solution, the solvent probably displacing the triazolopyrimidine ligand from the coordination sphere of the metal atom. This decomposition is almost complete after 24 hours.

*Crystal structure of* **1**

The molecular structure of **1**, depicted in Figure 1, is almost identical to that of the analogous compound with the ligand HmtpO[20] which differs of 7HtpO by the presence of a methyl group at position 5. Both compounds are not, however, crystallographically isostructural.

The platinum atom is in a typical square-planar environment (see distances and angles in Table 2), coordinated by two chloride anions and two N3-bonded 7HtpO ligands in a cis disposition. The relative orientation of the ligands is head-head, stabilized by the presence of a water molecule (O1W) which acts as acceptor of hydrogen bonds of the N4-H groups of both ligands. The second water molecule also contributes to the stabilization of the complex by the formation of hydrogen bonds with O1W and one of the chloride ligands.

Table 2. Selected distances (Å) and angles (1) for **1**

| Bond distances | | Bond angles | |
|---|---|---|---|
| Pt-Cl1 | 2.298(2) | Cl1-Pt-Cl2 | 92.10(7) |
| Pt-Cl2 | 2.278(2) | Cl1-Pt-N3A | 91.7(2) |
| Pt-N3A | 2.025(7) | Cl1-Pt-N3B | 177.2(2) |
| Pt-N3B | 2.008(6) | Cl2-Pt-N3A | 175.2(2) |
| | | Cl2-Pt-N3B | 88.4(2) |
| Hydrogen bonds | | N3A-Pt-N3B | 88.0(3) |
| N4A...O1W | 2.808(10) | | |
| N4B...O1W | 2.774(9) | | |
| O1W...O2W | 2.811(12) | | |
| O1W...O7B$^i$ | 2.751(8) | | |
| O2W...Cl2 | 3.093(9) | | |

See legend of Table 3.

*Antiparasitary tests*

The three triazolopyrimidine derivatives markedly affect the growth of the epimastigotes of *T. cruzi*, procyclic forms *T. b. brucei* and promastigotes of *L. donovani* cultured *in vitro*. The results are reflected in Tables 3, 4 and 5. This inhibition is highest for compound **1**. On the other hand, in the case of the growth of *P. characias* cells, **3** proved the most inhibitory, whereas **1** and **2** did not cause any significant growth inhibition, apparently due to the metabolic differences between the promastigote forms of *P. characias* and the other three trypanosomatids.

None of these derivatives presented cytotoxicity against macrophages of the J774.2 line (Table 6), at the heaviest dosages used (50 and 100 μM), in agreement with observations and studies by other authors for different pyrimidine derivatives.[21]

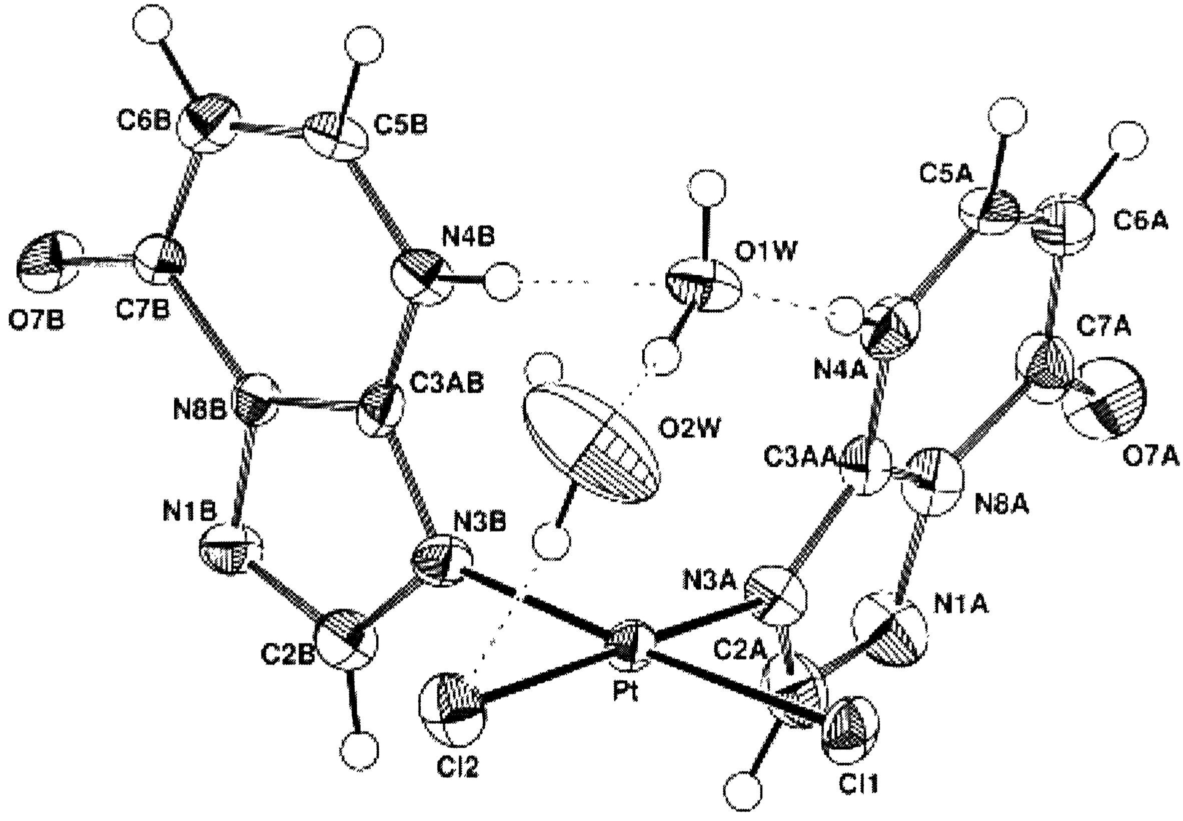

Figure 1 Molecular structure of compound **1**, as deduced from X-ray data. Non hydrogen atoms are shown as thermal ellipsoids at the 50% probability level.

TABLE 3: Percentage of growth inhibition caused by compound **1**.

| | [ 1 μM ] | | | [ 20 μM ] | | | [ 50 μM ] | | | [ 100 μM ] | | |
|---|---|---|---|---|---|---|---|---|---|---|---|---|
| | 24 | 48 | 72 | 24 | 48 | 72 | 24 | 48 | 72 | 24 | 48 | 72 |
| *T. cruzi* | 1 | 15 | 10 | 8 | 30* | 33* | 11 | **42**** | **54**** | 26 | **66**** | **81**** |
| *T. brucei brucei* | - | 18 | 37* | 16 | 39* | **47**** | 7 | **50**** | **74**** | 30* | **61**** | **85**** |
| *L. donovani* | 12 | 25 | 37* | 16 | 36* | **47**** | 38* | **48**** | **56**** | **52**** | **62**** | **94**** |
| *P.characias* | - | - | 16 | - | - | 7 | - | 20 | 5 | - | 11 | 8 |

The results are expressed in percentages of cell-growth inhibition (n=5). Significant differences between control and treatment cells were found according to the Newman-Keuls test. * P< 0.05. ** P< 0.025.

TABLE 4: Percentage of growth inhibition caused by compound **2**.

| | [ 1 µM ] | | | [ 20 µM ] | | | [ 50 µM ] | | | [ 100 µM ] | | |
|---|---|---|---|---|---|---|---|---|---|---|---|---|
| | 24 | 48 | 72 | 24 | 48 | 72 | 24 | 48 | 72 | 24 | 48 | 72 |
| *T.cruzi* | 15 | 12 | 18 | 23 | 25 | 38* | 17 | 29* | **46**** | 30* | 39* | **59**** |
| *T. brucei brucei* | 10 | 13 | 19 | 7 | 17 | 38* | 8 | 21 | **48**** | 21 | **50**** | **70**** |
| *L. donovani* | 7 | 15 | 32* | 7 | 18 | 36* | 22 | 35* | **56**** | 32* | **45**** | **74**** |
| *P. characias* | - | - | - | 10 | - | 2 | 10 | - | 21 | - | - | 25 |

TABLE 5: Percentage of growth inhibition caused by compound **3** (See legend of Table 3).

| | [ 1 µM ] | | | [ 20 µM ] | | | [ 50 µM ] | | | [ 100 µM ] | | |
|---|---|---|---|---|---|---|---|---|---|---|---|---|
| | 24 | 48 | 72 | 24 | 48 | 72 | 24 | 48 | 72 | 24 | 48 | 72 |
| *T. cruzi* | 12 | 18 | 24 | 14 | 20 | 31* | 17 | 27* | **40**** | 28* | **43**** | **61**** |
| *T. brucei brucei* | 22 | 23 | 19 | 27* | 29* | 32* | 33* | 35* | **41**** | 36* | **46**** | **61**** |
| *L. donovani* | 8 | 11 | 16 | 14 | 15 | 34* | 27* | 33* | **43**** | 36* | **43**** | **59**** |
| *P. characias* | 3 | 9 | 25 | 14 | 22 | 29* | 15 | 29* | **48**** | 24 | 33* | **56**** |

TABLE 6: Action of the complexes against mouse macrophage J-7740 cell line (Percentage of cell viability).

| | 24 h. | | 48 h. | | 72 h. | |
|---|---|---|---|---|---|---|
| | 50 µM | 100 µM | 50 µM | 100 µM | 50 µM | 100 µM |
| Control | 76.29% | | 93.45% | | 82.88% | |
| **1** | 65.00% | 68.57% | 95.83% | 90.20% | 83.16% | 87.38% |
| **2** | 75.28% | 95.45% | 96.74% | 93.39% | 90.55% | 90.91% |
| **3** | 78.68% | 80.91% | 94.37% | 89.16% | 81.56% | 85.61% |

Number of experiments: 3.

### ACKNOWLEDGMENTS

This work was supported by a DGESIC project (PB 97-0786-CO3) from the *Ministerio de Educación y Cultura*. M. Abul Haj is grateful for a grant of the PEACE program.

### REFERENCES

1. Oliveira, M.M. Inositol Metabolism in *Trypanosoma cruzi*: Potential Target for Chemotherapy Against Chagas' Disease. *Third Internet Conference on Salivarian Trypanosomes and other Trypanosomatids*. 2000.
2. Bales, J.D., *Tropical Medicine and Emerging Infectious Diseases*. G.T. Strickland Ed. 7$^{th}$ ed, pp 617-628, Saunders, Philadelphia, 1991.
3. Camargo, E.P., Wallace, F.G., *Advances in Disease Vector Research* **10**, 333, 1994.
4. Croft, S.L., *Mem. I. Oswaldo Cruz*. **94**, 215, 1999.
5. Pepin, J., Milord, F., *Adv. Parasit*. **33**, 1, 1994.
6. Harmon, M.A., Scott, T.C., Li, Y., Boehm, M.F., Phillips, M.A., Mangelsdorf, D.J., *Exp. Parasitol.* **87**, 229, 1997.
7 Fischer, G., *Adv. Heter. Chem.* **57**, 81, 1993.
8. Chae, M.Y., Swenn, K., Kanugula, S., Dolan, M.E., *J. Med. Chem.* **38**, 359, 1995.
9 Baraldi, P.G., Cacciari, B., Spalluto, G., Villatoro, M.J., Zocchi, C., Dionisotti, S., Orgini, E., *J. Med. Chem*. **39** 1164, 1996.
10. Salas, J.M., Romero, M.A., Sánchez, M., Quirós, M., *Coord. Chem. Rev.* **193-195**, 1119, 1999.
11. Haasnoot, J.G., *Coord Chem. Rev* **200-202**, 131, 2000.
12. Navarro, J.A.R, Romero, M.A., Salas, J.M., Quirós, M., El Bahraoui J., Molina J., *Inorg. Chem* **35**, 7829, 1996.
13. Navarro, J.A.R., Romero, M.A., Salas, J.M., *J. Chem. Soc Dalton Trans.*, 1001, 1997.
14. Luque, F., Fernández-Ramos, C., Entrala, E., Rosales, M.J., Navarro, J.A.R., Romero, M.A., Salas, J.M, Sánchez-Moreno, M. *Comp. Biochem. Phys.* **C126**, 39, 2000.

15. Luque, F., Fernández-Ramos, C., Entrala, E., Rosales, M.J., Marín, C., Salas, J.M., Navarro, J.A.R., Sánchez-Moreno, M. *Toxicol. In Vitro.* **14**, 487, 2000.
16. Abul Haj, M., Salas, J.M., Quirós, M., Molina, J., Faure, R., *J. Mol. Struct.* **519**, 165, 2000.
17. Sheldrick, G.M. SHELXL 97. *Program for the Refinement of Crystal Structures.* University of Göttingen, Germany, 1997. Available at http://shelx.uni-ac.gwdg.de/SHELX/.
18. Ruiz-Pérez, L.M., Osuna, A., Castanys, S., Gamarro, F., Craciunescu, D., Doadrio, A., *Drug-Research.* **36**, 13, 1986.

19. Fernández-Becerra,C., Sánchez-Moreno,M., Osuna, A., Opperdoes, F.R., *J. Eukaryot. Microbiol.* **18**, 230, 1997.
20. Navarro, J.A.R., Salas, J.M., Romero, M.A., Vilaplana, R., González-Vílchez, F., Faure, R., *J. Med. Chem.* **41**, 332, 1998.
21. Baraldi, P.G., Cacciari, B., Spalluto, G., Villatoro, M.J., Zocchi, C., Dionisotti, S., Orgini, E., *J. Med. Chem.* **39**, 1164, 1996.

**Received: May 23, 2001 – Accepted: June 18, 2001 – Accepted in publishable format: June 19, 2001**

